# The study of immunological markers in tuberculosis across animal models and its translation to human research

**DOI:** 10.1038/s41684-026-01730-9

**Published:** 2026-06-04

**Authors:** Sergio Díaz-Fernández, Matilde Aleluia, Margarida Saraiva, Pablo Soldevilla, Jordi B. Torrelles, Riti Sharan, Frank A. W. Verreck, Angelo Izzo, Maria Vidal, Ana C. Moreira, Bernat Pérez de Val, Francisco Jose Roca, Madalina Preda, Eduard Torrents, Esther Julián, José Domínguez, Irene Latorre

**Affiliations:** 1 Institut d’Investigació Germans Trias i Pujol, Badalona, Spain; 2 CIBER Enfermedades Respiratorias, Instituto de Salud Carlos III, Madrid, Spain; 3 Genetics and Microbiology Department. Universitat Autònoma de Barcelona, Bellaterra, Spain; 4 i3S – Instituto de Investigação e Inovação em Saúde, Universidade do Porto, Porto, Portugal; 5 Population Health Program Southwest National Primate ResearchCenter, Texas Biomedical Research Institute, San Antonio, TX USA; 6 Host–Pathogen Interactions Program Southwest National Primate Research Center, Texas Biomedical ResearchInstitute, San Antonio, TX USA; 7 Section of TB Research and Immunology, Biomedical Primate Research Centre, Rijswijk, the Netherlands; 8 Centenary Institute, The University of Sydney, Sydney, New South Wales Australia; 9 Unitat mixta d’investigació IRTA-UAB en Sanitat Animal, Centre de Recerca en Sanitat Animal, Campus de la Universitat Autònoma de Barcelona, Bellaterra, Spain; 10 IRTA. Programa de Sanitat Animal, Centre de Recerca en Sanitat Animal, Campus de la Universitat Autónoma de Barcelona, Bellaterra, Spain; 11 Department of Biochemistry and Molecular Biology and Immunology Infectious Disease Pathology, Clinical Microbiology and Tropical Medicine, Biomedical Research Institute of Murcia (IMIB-Pascual Parrilla), University of Murcia, Murcia, Spain; 12 Department of Microbiology, Parasitology and Virology, Faculty of Midwives and Nursing, ‘Carol Davila’ University of Medicine and Pharmacy, Bucharest, Romania; 13 Clinical Laboratory of Medical Microbiology, Marius Nasta Institute of Pneumology, Bucharest, Romania; 14 Institute for Bioengineering of Catalonia, The Barcelona Institute of Science and Technology, Barcelona, Spain; 15 Microbiology Section, Department of Genetics, Microbiology and Statistics, Faculty of Biology, University of Barcelona, Barcelona, Spain; 16 Present Address: Integrated Research Center for Infectious DiseasesDivison of InfectiousDiseases Department of Internal Medicine College of Medicine, The Ohio State University, Columbus, OH USA

**Keywords:** Tuberculosis, Bacterial host response, Cytokines, Chemokines

## Abstract

Tuberculosis (TB), a disease caused by *Mycobacterium tuberculosis*, remains one of the major causes of death from infection worldwide, with over a million associated deaths each year. The study of biomarkers for TB is critical for advancing our understanding and management of the disease. Biomarkers, defined as measurable indicators of biological states or conditions, are invaluable for the diagnosis, prognosis and treatment monitoring of TB. Clinical studies have provided critical knowledge on the matter but are also notoriously constrained by economical, ethical and sampling limitations. The use of animal models provides a simpler, more controllable, cost-effective setting with great potential for translation to humans. They also allow the evaluation of biomarkers within the respiratory compartment, when available, which is of particular interest due to the nature of TB pathogenesis. This Review focuses on the current landscape of TB biomarker discovery in several animal models, from invertebrates to large mammals. Here we summarize the basics of host–pathogen immune interaction, describe the main methodological approaches used and highlight the most substantial findings for each animal model studied. Furthermore, we discuss the advantages, challenges and limitations associated with species-specific differences in animal models. We conclude that integrating the data obtained from animal models and human studies is absolutely required to advance the TB field to accelerate the management of this disease.

## Abstract (web summary)

This Review summarizes tuberculosis biomarker discovery across animal models, outlining immune mechanisms, methods and important findings, while stressing how integrating animal and human data is essential to advance tuberculosis diagnosis and management.

## Main

**Fig. 1:**
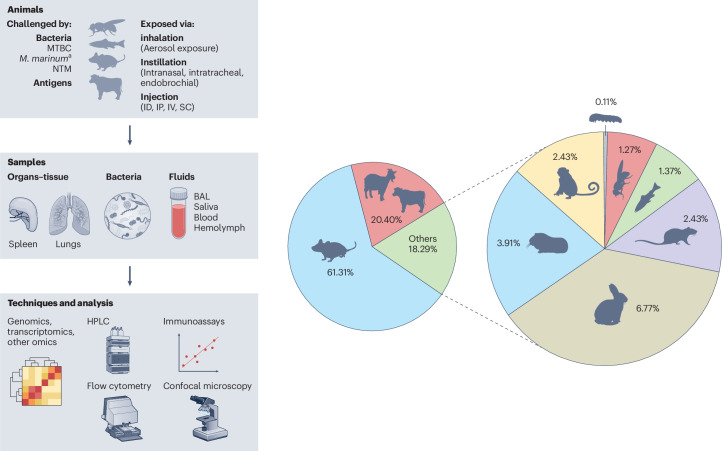
Flow diagram depicting typical pipelines in biomarker investigation in animal models and the proportion of each animal model in TB biomarker research. In TB-related studies, the animals are usually infected, vaccinated or challenged with mycobacterial species or antigens. Samples are usually collected from the pulmonary compartment or the spleen, but can also include lymph nodes, blood and any organ of interest. These will be processed to perform omics assays, flow cytometry, specific immunoassays and bacillary counts, among other tests. The percentage indicated for each animal model represents the proportion of articles for each model relative to the total of articles. The number of publications for each model was obtained from NCBI after the following MeSH search: (“tuberculosis”[MeSH Terms] OR “tuberculosis”[All Fields]) AND (“biomarkers”[MeSH Terms] OR “biomarkers”[All Fields]) AND (“X” [MeSH Terms] OR “X”[All Fields]), where X represents each animal name, namely: *Drosophila melanogaster*, galleria, zebrafish, mice, rat, guinea pig, rabbit, bovine OR caprine, NHP. ^a^*Mycobacterium marinum* is separated from the rest of the NTM because it is often used as a surrogate for Mtb, while most other NTM are used to study their specific pathologies. MTBC, Mtb complex (bacteria taxon that includes Mtb*, M. bovis*, *M. caprae*, *M. microti*, *M. africanum* and others); IV, intravascular; IP, intraperitoneal; ID, intradermal; SC, subcutaneous; BAL, bronchoalveolar lavage; HPLC, high-performance liquid chromatography.

Tuberculosis (TB) remains one of the leading causes of death worldwide. In 2023 alone, an estimated 10.8 million people were infected with the causative agent of the disease, *Mycobacterium tuberculosis* (Mtb), and approximately 1.25 million people died from the disease^1^. A better understanding of the complex host–Mtb relationship is needed to develop novel vaccines, improve current TB therapies and detect complex disease stages among latent TB (LTB) and active TB disease. Thus, it is important to identify and validate immune correlates of protection (COP) and disease^2^, with biomarker discovery being an intrinsic part in this research. A biomarker is defined as “any substance, structure, or process that can be measured in the body or its products and influence or predict the incidence of outcome or disease”^3^, and this measurement has to be quantifiable in an objective manner, regardless of the clinical state or sense of well-being^4^. Biomarkers can be classified according to their putative use as follows: susceptibility/risk (potential for developing disease), drug safety, predictive (likelihood of experiencing favorable or unfavorable events after exposure), diagnostic (detection of disease), prognostic (likelihood of disease progression), treatment response and monitoring (serial assessment of the disease)^5^. Many biomarkers meet the criteria for different uses; thus, definitions may overlap. We will focus this Review on those that could be useful for diagnosis (reflecting infection and disease status) and for vaccination and therapeutic efficacy, as these biomarkers have been used in many clinical studies over the past two decades^6^. However, clinical research is hampered by time, space, ethical and especially methodological restrictions associated with human studies. Animal models are used to overcome these difficulties, at least partially. In fact, the study of TB biomarkers has greatly benefited from animal experiments owing to the strict control of experimental conditions and the simplicity of the experimental units^7^. Animals can be challenged and monitored, and their organs can be analyzed in a process that ranges from a few weeks to several months (Fig. 1). In this Review, we aim to describe the state of the art in biomarker discovery within the context of animal work in TB and their contribution to the characterization of TB in humans. After a brief explanation of the immune response against Mtb and the limitations of human studies, this Review will provide detailed information on the main findings in immune biomarker research in the following animal models, as listed: fruit flies, greater wax moth, zebrafish, mouse, rat, guinea pig, rabbit, bovine and caprine, and nonhuman primates (NHPs). This Review also includes a last section with biomarker study for nontuberculous mycobacteria (NTM).

## Immune response against Mtb

Deeper knowledge is needed to comprehend the crosstalk between Mtb and the host, as TB induces a broad spectrum of immune responses and outcomes. In immunocompetent humans, for example, approximately 10% of individuals will develop disease with different manifestations after infection, generally occurring within the first 2 years after Mtb exposure^8^. Nowadays, it is difficult to identify the stages within the spectrum of TB disease, or even to predict whether a Mtb infection will result in disease development, which heavily depends on complex interactions between the innate and adaptive immune response and the bacterium^9–11^. Studying Mtb–host interactions in vivo using animal models has helped uncover many key components of the human immune response against Mtb. Together with human studies, these animal models have helped to reconstruct the sequence of events from the initial Mtb–host encounter to subsequent clearance, containment or progression of the infection.

### Innate immunity

Much of our understanding of the innate immune response in TB (extensively reviewed elsewhere^12,13^) comes from studies using experimental models of Mtb infection, most notably the mouse model. This is due not only to the many advantages of mouse models, but also to the fact that establishing the time of infection and accessing the local site of infection to study the initial immune events is technically and ethically challenging in humans. It is well established that the spread of Mtb occurs via aerosols or air droplets produced by individuals with disease and inhaled by a new host^14^. What remains unclear is the dose of bacilli required to establish a successful infection.

Experimental models have shown that, when Mtb reaches the alveoli of the lungs, it first interacts with soluble innate components of the lung mucosa^15^ and then with the first host cells to be infected: the resident alveolar macrophages (AMs)^16,17^. Studies performed in the past decade have enhanced our understanding of the role of AMs during Mtb infection^16,18,19^. Other cells are also thought to participate in the early events of infection, such as alveolar epithelial cells, which produce immunomodulators that contribute to the influx of other immune cells to the infection site^20^, and neutrophils, which release antimicrobial components and initiate inflammatory mechanisms in lung tissue^21^. Recruited dendritic cells (DCs), monocytes and other antigen-presenting cells take up bacilli, contributing to the development of the immune response to Mtb^22^. The diversity of these initial interactions is dictated by host-related factors, but also by the genetic variations of the pathogen itself^23,24^.

Several innate immune mediators have been commonly delineated as either beneficial or detrimental to the host in both mouse models and humans. Among these, neutrophils have been associated with poor prognosis of TB^25,26^, and a type I interferon (IFN)-inducible neutrophil signature has been associated with active TB disease^27^. Tumor necrosis factor (TNF) neutralization has been associated with reactivation of LTB^28^, and imbalances between the eicosanoids prostaglandin E2 (PGE2) and lipoxin A4 (LXA4) have been linked with resistance or susceptibility to disease^29,30^. A major challenge in the field remains not only unlocking but also harnessing innate immune events toward TB protection in humans.

### Adaptive immunity

The adaptive immune response to Mtb originates after T and B cell priming in the lung-draining lymph nodes, followed by the recruitment of primed cells to the site of infection for granuloma formation. Systemic T cell responses in humans can be detected by the tuberculin skin test (TST) 4–6 weeks post-infection. In mouse studies, the adaptive immune response is detected within 7–14 days after infection^31^. Despite its heterogeneity, the structure of the granuloma is mainly composed of macrophages on the inner layer, surrounded by granulocytes, T cells, B cells, natural killer (NK) cells and nonhematopoietic cells such as fibroblasts and epithelial cells. A major challenge in studying granulomas in preclinical animal models is their difficult characterization due to morphology diversity between models and humans^32^. Differences among animal models have helped describe the general structure of the granuloma and its role, as discussed through the model-specific approaches described in this Review.

Both CD4^+^ and CD8^+^ T cells are critical for the control and containment of Mtb, with IFN-γ-producing T cells having a central role. This has been indicated by the increased risk of TB associated with CD4^+^ T cell depletion in individuals infected with human immunodeficiency virus (HIV), before starting antiretroviral therapy^33,34^. Given that Mtb is an intracellular pathogen, T helper (Th)1 responses have an important role in host protection via macrophage activation and pathogen clearance. However, studies on TB vaccine candidates have shown that enhancing CD4^+^ T cells that produce IFN-γ or other Th1 cytokines does not necessarily correlate with increased protection in animal models and humans^35,36^. Therefore, future work needs to identify IFN-γ-independent mechanisms and biomarkers of protection or susceptibility. In this regard, Th17 responses, often accompanied by neutrophilic inflammation and higher tissue damage, have been closely linked to TB pathology. However, whether they have an active role in controlling the infection or are the result of Mtb escaping Th1 responses needs to be further investigated.

Mtb infection can also induce antibody responses, although the role of antibodies and B cells in bacterial pathogenesis and control is still unclear. Some studies have demonstrated the protective role of antibodies in humans and NHPs^37,38^. Antibody glycosylation patterns might also have an important role in immune responses^39,40^. Intriguingly, a study has shown that antibodies against Mtb are detected in individuals persistently exposed to Mtb, in the absence of IFN-γ-mediated T cell responses, which suggests an early protective role for B cell responses^41^. Altogether, it is important to note that no true COP against TB has been found because of the complex interactions among different immune components, which are not only limited to T cells.

## Specific challenges in human TB studies

The outcome of Mtb infection depends mainly on the ability of the immune system to clear or contain the pathogen. When this process fails, the bacteria begin to replicate, disseminate, and elicit inflammation, leading to disease. LTB diagnosis relies then on the evidence of cellular immune response against Mtb antigens, which is the basis of TST and the IFN-γ release assays^42^. However, these current immune diagnostic tests cannot discriminate between latent, incipient, asymptomatic, active and cured TB, nor predict the progression of latency to disease. Mtb infection is a term that has been evolving, and currently, it is known to encompass a spectrum of diverse states, which require different diagnostic and treatment approaches. Despite the challenges in distinguishing immunologically and microbiologically the different states within the TB spectrum, there is a consensus on unified criteria for their classification^43^: (1) uninfected; (2) Mtb infection; (3) incipient TB; (4) asymptomatic TB; (5) TB disease with positive microbiological findings and symptoms; and (6) past TB. It is important to note that there is still a considerable knowledge gap regarding biomarkers that can predict the progression of infection to early TB forms (incipient or asymptomatic TB) or disease. Studies targeting participants with incipient and asymptomatic forms require careful and exhaustive active case finding. If we also consider the high prevalence of comorbidities in human cohorts with TB, managing such studies becomes highly complex^44^.

Despite the high mortality associated to TB, it is well established that an early diagnosis of active TB and an accurate treatment can break Mtb transmission cycles and improve patient care and outcome^45^. However, several factors limit the control of the disease, such as the limitations in diagnostic methods for active TB, the emergence of resistant strains alongside challenges in drug-susceptibility testing and the lack of availability of point-of-care methods in high-burden settings. The World Health Organization recommendations for monitoring treatment response in adult pulmonary TB are sputum smear microscopy and/or culture conversion at the end of the intensive phase of TB treatment^46^. However, these methods have either suboptimal accuracy or a long turnaround time. The extensive heterogeneity in the design and reporting of treatment-monitoring studies is a major barrier to evaluating the performance of novel biomarkers in clinical trials and of tools developed for this use case^47^. Guidance on the design and reporting of treatment monitoring studies is urgently needed.

The most common site of TB is the lung, and the point of entry for Mtb is via the respiratory tract. For this reason, immunological biomarkers detected in the lungs might be of special interest to understand the immune responses to the infection; they may also more accurately reflect disease activity than those detected in peripheral samples. Preliminary studies have shown that biomarkers detected in response to mycobacteria are highly localized and, in some cases, absent from the peripheral blood^48^. A better understanding of the role of pulmonary-specific biomarkers may be of great relevance in the understanding and management of the disease. However, large studies exploring lung territories that may require invasive samples are not easy to perform in most TB clinical settings.

**Fig. 2:**
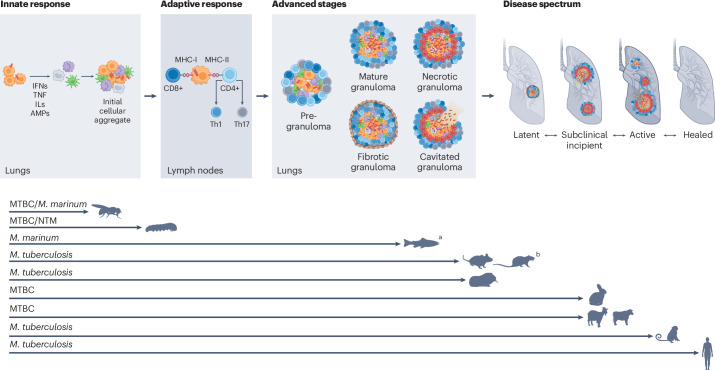
The contribution of each animal model to our understanding of the immune response against TB. The main pathogen used for each animal model to mimic TB infection (bottom), and the extent to which their immune response recapitulates the different stages of human disease (top). As can be observed, the non-mammalian models can provide information on the innate and adaptive responses, while larger, more complex animals can reproduce some features of latent or asymptomatic stages. ^a^Although zebrafish present necrotic granulomas, the presence of complex granuloma structures with cavitation has not yet been fully characterized. ^b^For mice, the large number of possible host and pathogen strains, infection routes and many other factors make it difficult to determine the exact contribution of this model regarding the information it can provide. MTBC, Mtb complex; MHC, major histocompatibility complex.

Therefore, there is a real clinical need for biomarkers that correlate with the risk of progressing from LTB to active TB, or help to characterize the different stages of the TB spectrum, predict the outcomes and also explore the TB–host interaction at the lung level^45^. This is where preclinical animal models can be very useful. However, the immune responses against Mtb are highly complex, and therefore no animal model can recapitulate all aspects of human TB disease. Instead, different animal models should be used, with simpler organisms providing information on the early innate responses and more complex models providing insights into the advanced stages of the disease and organ-specific responses (Fig. 2).

## Animal models for the study of biomarkers

This next section will cover the main invertebrate and vertebrate animal models used to study Mtb infection and TB disease, and the main advances in TB biomarker investigation that these models have provided. We will describe the advantages and limitations of each model (summarized in Table 1). Special focus will be given to mouse-based research, as this is the most studied animal model for experimental TB (see percentages in Fig. 1).

**Table 1:** Summary of advantages, limitations and main contributions to TB biomarker research of each animal model

Animal model	Advantages	Limitations	Contribution to the TB biomarker field
Greater wax moth (*G. mellonella*)	Small cost and space requirements No regulatory restrictions Similar body temperature to humans (kept at 37 °C) Easy manipulation	Lack of lungs and of adaptive response Short lifespan Lack of laboratory reagents	Not largely explored Great potential for the assessment of antimicrobial effectiveness
Fruit fly (*D. melanogaster*)	Small cost and space requirements No regulatory restrictions Easy availability of large populations Well-known genome	Lack of lungs and of adaptive response Short lifespan Infection with *M. marinum* and not Mtb	Study of AMP signature Knowledge of early mechanisms of defense against infection such as autophagy Initial studies before larger mammals
Zebrafish (*D*. *rerio*)	Small cost, moderate space requirements Opportunity to dissect innate and adaptive immune responses with larvae and adult life cycles, respectively Optical transparency allows easy visualization assays Genetically and pharmacologically tractable	Lack of lungs Infection with *M. marinum* and not Mtb Not suitable to study airborne transmission	Studies on macrophage migration, granuloma-associated blood vessels, necrosis of infected macrophages, granuloma formation and structure Identification and characterization of mycobacterial virulence factors Cytokine signature in response to *M. marinum* infection
Mice (*M. musculus*)	Moderate cost and space requirements Easiest mammal to handle Large immune toolkit High number of procedures described Similar lung structure and immune response to humans	Different granuloma to humans Most strains show low Mtb susceptibility Reproducibility depends on host and pathogen specificity	Study of trained innate immunity markers Study of highly specific, complex cell immune subsets Identification of lung resident cell markers associated with local protection Numerous cytokine, chemokine and associated molecule biomarker panels Cornell model is useful for studies of biomarkers of TB progression, but TB reactivation in mice is rarely natural
Rat (*R. norvegicus*)	Moderate cost and space requirements Easy animal to handle Larger organ sizes and blood volumes than mice	Most strains show low Mtb susceptibility Human pathology is not fully reflected as lesions do not liquify Least developed model of among vertebrate animals	Knowledge of drug efficacy and safety in anti-TB therapy Study of cytokine kinetics associated with granuloma and pathology Potentially useful to study latency
Guinea pig (*C. porcellus*)	Moderate space requirements Susceptible to very low-dose Mtb Similar granuloma to humans Fast development of the disease	Very limited immune toolkit Longer gestation than mice Lack of mutant strains	Markers of PBMCs and cytokine signatures associated with bacterial clearance after vaccination Study of organ-specific metabolites in blood Fast evaluation of vaccine efficacy and biomarkers associated to protection
Rabbits (*O. cuniculus*)	Relatively large body size with easily accessible organs Similar granuloma to humans Similar histopathology and hypoxic microenvironment to humans	Relatively limited immune toolkit Low Mtb susceptibility Moderate-to-high cost and space limitations	Useful for the discovery of biomarkers of TB progression from latency-like states to cavitary TB Study of markers in alternative, nonpulmonary forms of TB Evaluation of drug penetration depending on lesion type and severity
Bovine and caprine	High similar immunopathology and granuloma to humans Relatively high number of reagents available for immunoassays (especially for bovine, but many cross-reacting with goat) Long lifespan allows for long prospective studies Large blood volume and organ sizes	Large and expensive facilities for animal housing Requires specialized personnel No natural susceptibility to Mtb	Study of pro-inflammatory markers associated with severity of complex TB pulmonary lesions Study of trained innate immunity markers Study on early humoral markers associated with bacterial load The spectrum of disease outcomes makes these models valuable for the study of biomarkers of progression from asymptomatic to active TB
NHP (*M. mulatta,* *M. fascicularis, C. jacchus*)	Most similar anatomy and pathology to humans Model that best recapitulates features of human TB infection Relatively large immune toolkit Can be infected with SIV to study HIV/TB co-infection	Very high cost and space limitations Requires specialized personnel Ethical concerns	Study of trained innate immunity markers Study of spectrotypes within disease Evaluation of protective markers during latency stages and granuloma formation Ideal model for the study of biomarkers of TB progression and reactivation

### Invertebrate models

#### The fruit fly

*Drosophila melanogaster* (fruit fly) is probably the simplest and fastest animal model for the study of TB. Its immune system relies exclusively on innate immune responses, including both humoral and cell-mediated components, whose features are highly conserved with those of vertebrates^49,50^. The *D. melanogaster* humoral response is mainly driven by the production of two antimicrobial peptides (AMPs), drosomycin (via the Toll pathway, homolog to the mammal Toll/interleukin (IL)-1 one) and diptericin (via the immune deficiency pathway homolog to the mammal TNF receptor 1 (TNFR1) pathway)^51^. Phagocytosis represents a fundamental process of the innate cellular immune response in *D. melanogaster*. It is mainly performed by plasmatocytes, homologs of the vertebrates’ macrophages. These phagocytes also contribute to the humoral response via the expression of Unpaired (Upd) proteins, which activate Janus kinases and signal transducer and activator of transcription proteins (JAK/STAT) signaling pathways^50^. In addition, the immune response of the flies is also modulated by metabolic (insulin/insulin-like signaling (IIS)) and life-cycle hormonal (through the ecdysone steroid hormone) pathways^52,53^. In *D. melanogaster*, all these markers of immunity are used to characterize the pathogenic profile of the infection.

Mycobacterial infections in flies require the use of *Mycobacterium marinum*^54^ as a surrogate model of Mtb infection, which shares key virulence determinant and aspects of mycobacterial pathogenesis, such as the cytopathic effects in macrophages and epithelial cells, in ways that mirror essential aspects of the Mtb infection cycle. At the early stages of the infection, when *M. marinum* grows intracellularly in hemocytes, these cells strongly induce the expression of Upd3 (IL-6-like cytokine). Studies have demonstrated that Upd3 overexpression leads to a repression in the production of autophagy-related protein 2 (Atg2), favoring the replication and survival of *M. marinum* inside the plasmatocytes and thereby shortening lifespan of the host^55^. As the infection progresses, mycobacteria start growing both intra- and extracellularly, and AMPs are strongly induced. However, the type of AMP induction seems to differ depending on the sex and reproductive status of the flies. Indeed, reproductively active flies have a high production of diptericin (Imd pathway activation) upon *M. marinum* infection, which correlates with the basal levels of ecdysone receptor of those flies. Conversely, flies with no reproduction opportunities show production of drosomycin via Toll pathway activation^53^. Just as these immune markers can describe the infection process, they can also monitor treatment success. Some studies have shown the importance of autophagy in the efficacy of anti-TB drugs and highlighted Atg8a as a good marker of the pathology induced by mycobacterial infection^56,57^. Although biomarkers of infection in *D. melanogaster* have not been extensively explored, this model provides great insights into the early mechanisms of defense against infection in a rapid and cost-efficient manner. Because gene expression in *D. melanogaster* can be studied across any biological process or experimental condition, further work in the TB model will help to elucidate key aspects such as infection progression under specific conditions, pathogen virulence and treatment efficacy.

However, the simplicity of this model—reflected in its short lifespan, lack of an adaptive immune response, and absence of a pulmonary system—is also a double-edged sword. Together with the fact that the infection is performed with *M. marinum* and not Mtb, these limitations make it difficult to draw conclusions that can be directly extrapolated to more complex systems. Nevertheless, results obtained with this model will be useful for scaling to larger mammal animal models before translation to humans.

#### Greater wax moth

In the past 10 years, *Galleria mellonella* (greater wax moth) larvae have emerged as a surrogate model for infection studies and the assessment of antimicrobial effectiveness. Unlike other invertebrate models, *G. mellonella* has a more convenient size for manipulations and can be studied at temperatures up to 37 °C, mirroring the conditions of the human body. In addition, the greater wax moth retains the advantages of invertebrate models, such as its economical breeding and housing, and the avoidance of regulatory and ethical restrictions. Notably, its innate immune system is complex, sharing multiple similarities with that of humans, comprising both cellular and humoral defenses. The cellular response involves six types of hemocyte^58^, some of them analogous to mammalian neutrophils and macrophages, which can phagocyte and kill pathogens, by generating reactive oxygen species. Nodulation, encapsulation and the formation of neutrophil extracellular traps are further anti-infective functions of *G. mellonella* hemocytes. Following the activation of the host pattern recognition receptors through the Toll and Imd pathways, the humoral response leads to the release of AMPs, reactive oxygen/nitrogen species and hydrogen peroxide. Moreover, the melanin-based immune response is considered comparable to the complement cascade in mammals. These properties make this insect a comprehensive and appealing model for investigating mycobacteria infections.

As early as a century ago, reports described the capacity of *G. mellonella* hemocytes to phagocyte mycobacteria and induce the formation of granuloma-like structures^59^. In the past 7 years, diverse NTM have been studied, revealing that infection with pathogenic species such as *M*. *marinum*, *Mycobacterium fortuitum* or *Mycobacterium abscessus* results in dose-dependent larvae mortality^60–63^, while infection with nonpathogenic mycobacteria such as *Mycobacterium aurum* or *Mycobacterium brumae* does not affect larvae survival^61,64^. Few studies have used Mtb complex members in *G. mellonella*, and most of this work was done in the laboratory of Sandra M. Newton and Paul R. Langford^59,65–67^. Notably, these studies have shown that survival rates during *Mycobacterium bovis* Bacille Calmette–Guérin (BCG) infection are dose dependent^64–66^. A study comparing similar infection conditions for two Mtb strains showed that BCG forms intracellular lipid inclusions, leading to a nonreplicative and persistent infection within granuloma-like structures, while Mtb H_37_R_v_ replicates inside hemocytes, disseminates from the more abundant and larger induced granuloma-like structures and stimulates further immune response^59^. Regarding biomarker detection in mycobacteria-infected larvae, label-free quantitative proteomic analysis has identified both BCG and larvae-related proteins in hemolymph samples, indicating a unique immune profile over the course of infection^67^. Similarly, the analysis of the mRNA expression of innate immune-related genes in BCG- or *M. abscessus*-infected larvae has highlighted the relevance of AMPs or opsonizing agents in the host innate response^63,67^. Overall, these findings establish *G. mellonella* as a valuable model for mycobacteria infection studies, complementing data obtaining from other ex vivo and in vivo models.

A major limitation of this model is that, as with *D. melanogaster*, the larvae have a short lifespan (under optimal conditions, 8–10 weeks), which can interfere with the study of chronic infection processes or the slow bacterial division time. *G. mellonella* also lack pulmonary organs like the lung. Moreover, the extent of laboratory reagents available to study biological processes in *G. mellonella* is rather limited; as the toolkit expands, the more useful this model will become.

### Zebrafish

The zebrafish (*Danio rerio*) is the nonmammal vertebrate model with the most impact on TB research. The zebrafish contains both cellular effectors (macrophages, neutrophils and NKs, innate lymphoid and DCs) and humoral components (cytokines, chemokines, IFN and complement) that mediate an innate immune response with conserved functions compared with their mammalian counterparts^68^. The zebrafish also presents the cellular mediators required to develop an adaptive immune response (T and B cells and an array of molecules and tissues/organs)^69,70^, which becomes active when the animal is 30 days old. These characteristics have been very useful to study the different stages of the innate granuloma formation.

Similar to fruit fly, the main tuberculous zebrafish model involves infection with *M. marinum*, which in cold-blooded animals causes necrotizing granulomas similar to those observed with TB. Thus, this model can be considered as a surrogate to model the human disease^71^. From the host perspective, the zebrafish is easily tractable both genetically and pharmacologically and has the additional advantage of being optically transparent during the early stages of life, allowing host–pathogen interactions to be visualized in live animals in real time^72^. Notably, this transparency enabled the visualization of *M. marinum* trafficking within the macrophages that were recruited to the granulomas and the subsequent migration of such infected macrophages to new tissues during early tuberculous infection^73^. The larval zebrafish has also been used to study the association between angiogenesis (production of new blood vessels from pre-existing vessels) and granuloma formation. Indeed, a zebrafish study demonstrated that the macrophage levels of vascular endothelial growth factor (VEGF), a mediator of host vascularization whose expression is induced in human pulmonary TB^74^, can serve as marker of angiogenesis, and its inhibition reduces infection burden and mycobacteria dissemination^75^.

The ease of preparing organs or even whole bodies in zebrafish experiments has also facilitated the analysis of transcript and protein levels in both larval and adult stages. In larvae, transcriptome and infection burden analyses have identified *Tlr2* as a protective factor against mycobacteria^76^, IL-16 expression as a marker of pathology severity^77^ and *cxcl11* gene as a marker of macrophage polarization during infection^78^. Studies with adult zebrafish have also analyzed the differential expression of cytokines during infection. In this regard, mRNA levels of *tnf*, *ifn-γ*, *il-1β* and *mmp13* (matrix metalloproteinase gene, involved in granuloma formation) were upregulated in zebrafish liver and spleen after infection with *M. marinum* and correlated with pathology and mycobacterial load, suggesting that the upregulation of pro-inflammatory cytokines could serve as a marker of disease progression^79^.

The use of the zebrafish model has led to the identification and characterization of many bacterial factors involved in mycobacterial dissemination, including *zmp1* or the regulatory gene *whiB6*, and in bacillary survival, such as *mimG* encoding a mycobacterial phosphoribosyltransferase^80,81^. Of special importance is the ESX set of genes. *M. marinum* mutants lacking the *esx-1* secretion locus (essential for full virulence of Mtb) were associated with decreased granuloma formation and increased dissemination compared with peripheral tissues^82^. The importance of this virulence factor on Mtb dissemination was further corroborated in the mouse model^17^ and in human studies.

Despite the many advantages of the zebrafish model, it is still a lungless organism, and studies have typically used the surrogate *M. marinum* strain. Therefore, it is important to always confirm the findings in a larger vertebrate model or with the actual human pathogen.

### Small mammals: mice

The most used animal model to study all aspects of TB is the mouse (*Mus musculus*)^83^. Mice offer many advantages as an animal model to study TB: they are the cheapest mammal model to maintain, are easy to handle for different procedures, are susceptible to Mtb infection^84^ and present a remarkable degree of similarities with humans in terms of lung immunological characteristics and mechanisms^85^. Although most mouse strains can be infected with Mtb and eventually die from the infection, their general susceptibility to Mtb is rather low, with high strain-dependent heterogeneity. Mouse strains can be classified as ‘resistant’ and ‘susceptible’, with genetic differences driving this variability^86^. For this Review, only the main inbred mouse strains and novel models will be considered. C57BL/6 and BALB/c are the two most frequently used TB-resistant mouse strains. Their immune responses to mycobacteria are different, with the C57BL/6 strain showing stronger Th1 and early inflammatory responses^87^, while the BALB/c strain is Th2-skewed^88^. However, both strains develop chronic TB upon high-dose Mtb infection, rely on AMs and lymphocytes as the main containment response, and form granulomas with absent to low necrosis. Conversely, C3HeB/FeJ mice are considered the TB-susceptible reference strain, because they develop pulmonary necrotic lesions that can break down into a semiliquid/liquid debris (rich in lipids, matrix fragments and dead immune cells), which creates a microenvironment similar to that seen in advanced human disease. Their lungs also resemble those from humans in terms of hypoxia or caseation, but do not usually show classical granulomas^89^. The immune cell response of this FeJ strain is characterized by neutrophilic infiltration in the lungs, with a Th17-prominent response that ends with uncontrolled pulmonary inflammation and bacterial replication^90^. Due to these characteristics, this model is considered the best model for acute TB disease studies. The heterogeneity observed in mouse strain responses to infections has created opportunities to identify immune molecules associated with the progression of the disease, both in the periphery and in the lung, as described below.

#### Peripheral biomarkers

What arouses most interest in the biomedical field is the identification of indicators of progression from Mtb infection to disease using non-invasive samples such as urine or saliva. Although reports have indicated the presence of immunoglobulins IgM^91^ and IgA^92^ in mouse saliva after intranasal immunization, the extraction of these fluids generally yields little to no volume; therefore, only a few mouse studies have focused on such tissues, while most studies have instead focused on peripheral blood. In mice, peripheral blood extraction via nonterminal procedures is a relatively easy procedure that enables collection at different time points. However, there is limited literature on immune protein biomarkers identified in mouse blood samples that have subsequently been applied as potential biomarkers in humans. Conversely, there are examples of potential biomarkers first found in humans that have been subsequently confirmed in the mouse model, such as IL-10^93^ and CXCL-1^94^. In such cases, investigating the function of these proteins in the mouse model provides the opportunity to unravel their action mechanism(s). Another example is the detrimental roles of type I IFN and neutrophilic inflammation, which were first identified as part of a blood transcriptomic signature in human TB^27^ and were subsequently validated by lung RNA sequencing analyses using C3HeB/FeJ mice aerogenically infected with Mtb HN878^95^. The latter study also used blood transcriptomics to demonstrate the importance of granulocyte–macrophage colony-stimulating factor (GM-CSF) signaling in Mtb containment, showing that impaired GM-CSF signaling leads to increased mycobacterial growth and worsened lung pathology. Other research groups have used blood from Mtb-infected animals to identify specific RNA signatures that correlated with the progression of the infection and vaccine efficacy in mice^96,97^.

Other studies on murine blood transcript biomarkers have focused on microRNAs (miRNAs). miRNAs are small noncoding RNAs that regulate the expression of genes and that are found in most tissues and fluids, a feature that makes them ideal candidates as disease biomarkers^98,99^. Most studies focusing on murine miRNAs in the context of TB have been performed in C57BL/6 mice. The most prominent miRNA in TB studies is miRNA-223, which is abundantly present in the blood of mice infected with Mtb and the blood of patients with TB^100^. miRNA-223 was also shown to have a key role in protection against Mtb infection, as miRNA-223-deficient mice succumbed to infection substantially faster than wild-type (WT) mice. Conversely, in humans, a study found that miRNA-223 levels correlated with Mtb survival in patients with TB, by blocking macrophage apoptosis via FOXO3^101^. For further literature on the roles of miRNAs in TB, we refer readers to other sources^102^.

#### Pulmonary biomarkers

**Fig. 3:**
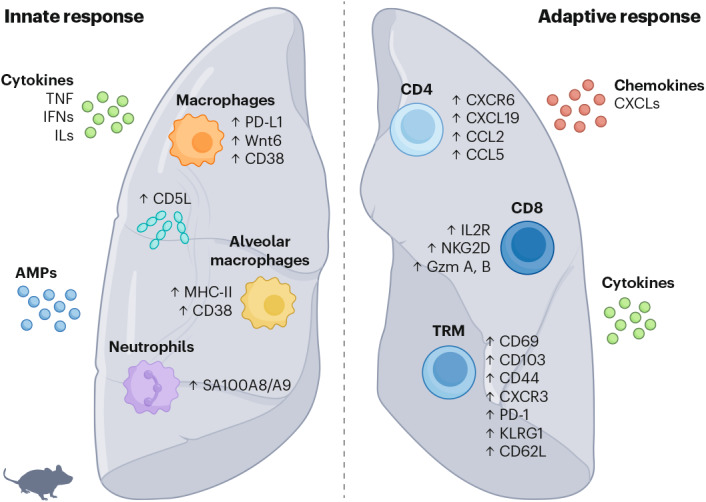
Main biomarkers within the murine pulmonary environment that may be associated with infection or protection against Mtb. Representation of the main murine pulmonary biomarkers associated with Mtb described in the text, with a visual indication of their main cells of origin and separation into innate and adaptive responses. NKG2D, NK group 2 member D, Gzm, granzyme.

Because systemic immunity may not reflect local responses in the pulmonary space, most of the studies in mice have focused on lung samples^48^. The early lung transcriptomic studies had already identified signature profiles in response to Mtb infection, with a wide array of proteins making compelling candidate biomarkers (see Gonzalez-Juarrero et al.^103^), including IFN-associated genes, chemokines, cytokine and activation markers. Below, we present a brief but comprehensive network of biomarker candidates, with insights into their relationships with different stages and types of immune responses against Mtb. This information is summarized in Table 2 and Fig. 3.

**Table 2:** List of TB biomarkers identified in mice

Biomarker	Type of molecule	Cell/tissue of origin	Mouse strain	Potential use^a^	Infection/vaccination/ stimulation status	Change	Reference
miRNA 223	miRNA	Lung tissue homogenate and blood	C57BL/6J	Diagnostic	Aerosol infection with Mtb H_37_R_v_	Increase associated with infection	Dorhoi et al.^100^
RNA signature	RNA transcriptional profile	Blood	C57BL/6J	Disease status	Aerosol infection with Mtb H_37_R_v_	Specific transcriptomic pattern for early and late infection	Ault et al.^96^
RNA signature	RNA transcriptional profile	Blood	C57BL/6J DO	Vaccine efficacy	Aerosol infection with Mtb H_37_R_v_	Specific transcriptomic pattern associated with vaccine efficacy	Kurtz et al.^145^
PD-L1	Gene, protein	BMDM	C57BL/6J	Diagnostic	Stimulation with heat-killed Mtb H_37_R_v_	Increase associated with infection	Pan S^105^
MHC-II	Surface protein	AM	ccr2^−^C57BL/6J	Vaccine efficacy	Intranasal Ag85 immunization, intranasal infection with Mtb H_37_R_v_	Increase associated with protection	D’Agostino et al.^106^
CD38	Surface protein	Macrophages, AM	C57BL/6J	Vaccine efficacy	Intranasal BCG immunization, intranasal infection with Mtb Erdman	Increase associated with early control of Mtb growth	Pisu et al.^107^
CD5L	Protein	Blood, lung tissue	C57BL/6	Diagnostic	Aerosol infection with Mtb HN878	Increase associated with infection	Cardoso et al.^108^
Wnt6	Protein	Mtb-infected macrophages	C57BL/6	Disease status	Aerosol infection with Mtb H_37_R_v_	Expression only in granulomatous lesions	Schaale et al.^109^
SA100A8/A9	Surface protein	Neutrophils	DO	Treatment efficacy	Aerosol infection with Mtb H_37_R_v_	Increase associated with pathology, decrease with treatment	Gopal et al.^110^
CXCR6	Chemokine	T cells	C57BL/6, BALB/c	Vaccine efficacy	Ad85, rec85A, ESAT6 immunization, intranasal infection with Mtb Erdman	Increase associated to protection	Lee et al.^123^
CCL2, CCL5, CCL7, CCL12, CCL19, CXCL9	Gene	Lung tissue homogenate	C57BL/6	Treatment efficacy	Aerosol infection with Mtb Erdman. When treated; INH, PZA, EMB delivery ad libitum	Increase in absence of treatment	Park et al.^124^
CXCL1, CXCL2, CXCL5	Chemokine	Lung tissue homogenate	DO, C57BL6/6J	Susceptibility	Aerosol infection with Mtb Erdman	Increase associated with Mtb susceptibility	Niazi et al.^126^
CXCL1, CXCL2, TNF, IL-10, MMP8	Protein	Lung tissue homogenate	C57BL/6, DO	Susceptibility	Aerosol infection with Mtb Erdman	Increase associated with Mtb susceptibility	Koyuncu et al.^94^
CXCR3^+^CX3CR1^−^ KLRG1^−^	Surface protein	Lung-resident CD4^+^ T cells	C57BL/6 KOs, CB6F1	Vaccine efficacy	Aerosol infection with Mtb H_37_R_v_ and Erdman	Increase associated with protection	Sakai et al.^119^ Sallin et al.^121^ Woodworth et al.^120^
IL-17a	Cytokine	CD4^+^ T cells	C57BL/6	Vaccine efficacy	Subcutaneous BCG vaccination and Mtb Erdman infection	Increase associated with protection	Derrick et al.^129^
IL-6, IL-11	Cytokine	Lung cell culture	C57BL/6	Disease status	Intravenous infection with Mtb H_37_R_v_	Increase associated with chronicity of infection	Kondratieva et al.^130^
IL-21	Cytokine	Lung homogenate	C57BL/6J	Susceptibility	Aerosol infection with Mtb Erdman	Increase associated with resistance	Booty et al.^131^
IFN-γ, IL-2, TNF	Cytokine	Mtb-specific CD4^+^ T cells	hSIGN, (C57BL/6 background)	Vaccine efficacy	Intravenous administration of Ag85B-overexpressing BCG	Increase associated with protection	Velasquez et al.^132^
IL-12p40	Cytokine	CD103^+^ DCs	C57BL/6, BALB/c	Diagnostic	Aerosol infection with Mtb Harlingen, H_37_R_v_	Increase associated with infection	Leepiyasakulchai et al.^134^
IL-2	Cytokine	KLRG1^−^ Mtb-specific CD4^+^ T cells	C57BL/6	Vaccine efficacy	Subcutaneous vaccination with BCG, aerosol infection with Mtb Erdman	Increase associated with protection	Lindenstrom et al.^114^
CD27	Surface protein	IFN-γ^+^ CD4^+^ T cells	C57BL/6	Disease status	Intratracheal/intravenous infection with Mtb H_37_R_v_	Decrease associated with lower severity	Kapina et al.^135^
Foxp3^+^, RoRγT^+^, IL-17	Protein	CD4^+^ T cells	CC	Vaccine efficacy	Subcutaneous BCG vaccination and Mtb H_37_R_v_ infection	Increase associated with protection	Lai et al.^143^
T-bet	Protein	CD4^−^CD8^−^ T cells	CC	Vaccine efficacy	BCG vaccination	Decrease associated with protection	Lai et al.^143^
CXCL2/MIP-2	Chemokine	Monocytes, macrophages	CC	Vaccine efficacy	Aerosol infection with WT and mutants of Mtb H_37_R_v_	Increase associated with protection	Smith et al.^141^
CCL3/MIP-1a	Chemokine	Neutrophils, macrophages	CC	Susceptibility	Aerosol infection with WT and mutants of Mtb H_37_R_v_	Increase associated with protection	Smith et al. ^141^
G-CSF	Growth factor	Macrophages	CC	Susceptibility	Aerosol infection with WT and mutants of Mtb H_37_R_v_	Increase associated with protection	Smith et al.^141^

The references shown are only the ones that are mentioned in the text and do not represent the entirety of bibliography on the matter.

^a^Some biomarkers can meet the criteria for different uses in preclinical stages; thus, definitions may overlap.

G-CSF, granulocyte colony-stimulating factor; BMDM, bone marrow-derived macrophage; hSIGN, human signaling lymphocyte activation molecule; INH, isoniazid; PZA, pyrazinamide; EMB, ethambutol.

##### Innate immune biomarkers

The initial stages of Mtb infection involve immune cell types classically implicated in infections with intracellular pathogens, mainly DCs, AMs and NK cells, although other immune cell types such as neutrophils have a key role in Mtb clearance and antigen presentation^104^. The predominance of pro-inflammatory molecules expressed by these innate cells provides an opportunity to find early-stage markers of TB protection. Some examples include the upregulation of programmed death ligand 1 (PD-L1) in lung macrophages after stimulation with heat-killed Mtb bacilli^105^, the upregulation of major histocompatibility complex class II (MHC-II) on AMs from T-cell-depleted mice after vaccination with Mtb antigen 85A^106^, the increase of CD38^+^ macrophages following BCG immunization intranasally^107^, the upregulation of local and systemic CD5L in Mtb-infected mice^108^, or the presence of wingless-type MMTV integration site family member 6 (Wnt6) protein in Mtb-infected macrophages as a potential biomarker for the generation of granulomatous lesions^109^. However, the most promising innate inflammatory marker for TB disease is S100 calcium-binding protein A8/A9 (SA100A8/A9) alarmin expression by neutrophils. This protein has a critical role in leukocyte recruitment and cytokine secretion, and mouse studies have shown fluctuations in pulmonary neutrophil-derived S100A8/A9 protein levels related to Mtb infection and drug treatment status^110^. This biomarker was more deeply studied in a Cornell model of disease reactivation after antibiotic treatment withdrawal. The findings showed lower levels of neutrophil accumulation, although with unaltered inflammation, in the granulomas of *S100A9*-knockout versus C57BL/6 mice. These results suggest that, while the protein might be involved in Mtb control, its expression is not related to the regulation of inflammation during TB^111^. The SA100A8/A9 biomarker has also shown interesting results in human studies, in both blood (mRNA levels indicating TB progressors, nonprogressors and drug-treated individuals^111^) and lung (predominance of S100^+^ neutrophils within inflammatory granulomas in patients with TB^110^) samples.

##### Adaptive immune biomarkers

The generation of Ag-specific, Th1-polarized T cells is usually considered the starting point for the adaptive immune response against TB. Given that CD4^+^ T cells are the hallmark of the TB immune response, it is not surprising that most mouse and human studies have focused on this cell type. Notably, most studies have focused specifically on the characterization of activation (PD-1, CD69 and CD44) and differentiation (killer cell lectin like receptor G1 (KLRG1) and CD62L) markers in lung Mtb-specific CD4^+^ T cells as indicators of TB disease severity or protection^112–115^. Although literature on CD8-related biomarkers for TB is scarcer, these cells also have a role in Mtb control; Cornell models of TB have notably shown their importance in maintaining TB latency^116^. One study focused on the role of CD8^+^ T cells in promoting the survival of Mtb-infected mice; the findings identified several biomarkers from the analysis of CD8^+^ T cell gene expression when cytotoxic pathways were enhanced by the presence of CD4^+^ T cells, including NK receptors *Nkg2d* and *2B4*, activation markers (*Il2ra* and *Cd69*) and cytotoxic molecules (*GzmA* and *GzmB*)^117^.

Currently, most attention in the TB field is focused on lung tissue-resident memory cells (TRMs), a subset of memory T cells persisting in the lungs without recirculation through the body. Because of their location, it is believed that lung TRMs represent an essential aspect of protective immunity to Mtb; however, the study of this cell population is hindered by the high vascularization of the lungs. Mouse models have provided the opportunity of using in vivo intravascular staining for the specific identification of TRMs^48,118^. The use of this technique has revealed that CD4^+^ T cells from the lung parenchyma, defined by high expression of CD69, CD103, PD-1 and CXCR3 residency markers, provided superior protection to Mtb compared with CD4^+^ T cells from the vasculature^119^. This is particularly interesting because CD4^+^ T cells in the lung vasculature express high levels of CX3CR1 and KLRG1, indicative of poor tissue migration and high IFN-γ production (a cytokine intrinsically associated with the immune response to TB), yet are associated with limited protection, further highlighting the importance of cellular localization in immunity^119–121^. Moreover, PD-1 was associated with an important role in protection by suppressing IFN-γ production in these lung vasculature cells^122^. Because most chemokines have a role in mobilizing blood and mesenteric immune cells into the lung parenchyma, they are also considered a feature of tissue residency. In this context, the expression levels of chemokine receptors CXCR6^123^ and CXCR3^119–121^ on lung T cells have been defined as COP against Mtb in C57BL/6 mice. Notably, chemokines are also useful for defining specific TB disease stages; for example, *Ccl2*, *Ccl5*, *Ccl7*, *Ccl12*, *Ccl19* and *Cxcl9* expressions expressions being increased in a chronic TB versus chemotherapeutic persistent stage in a TB infection model^124^ in C57BL/6 mice. The presence and activity of TRMs in human lungs have also been reported, with the analysis of bronchoalveolar lavage samples from patients with TB suggesting a critical role in local mucosal immunity^125^. However, much work is needed to determine the specific contribution of TRMs in human immunity against Mtb.

#### Released cytokines

Even though IFN-γ production by CD4^+^ T cells was for decades considered a hallmark of the immune response to TB, its potential as a TB biomarker is rather limited, as it is produced in response to most pathogens. IFN-γ levels or the number of Mtb-specific IFN-γ-producing cells in the lung directly reflect Mtb load but weakly correlate with TB disease progression^126^ or strength of protection^127^. Other so-called canonical cytokines in the TB immune response (TNF, IL-10 and IL-12p40) show high variations in their expression level between infections with different Mtb strains^128^, possibly due to the versatile nature of the mechanisms triggering the production of these molecules. Many other ILs have also been analyzed. For example, IL-17 expression correlates with protection after intranasal BCG vaccination^129^; IL-6 and IL-11 are substantially more secreted by C57BL/6 mouse lung cells compared with susceptible mouse strains after Mtb infection^130^; and pulmonary IL-21 levels increase with the recruitment of T cell into the lung 3 weeks after Mtb infection^131^. In the end, despite the existing body of literature, there is still no consensus on which cytokine can serve as a hallmark biomarker. This situation also applies to human studies, where the expression of pro-inflammatory cytokines alone is not sufficient for proper TB disease stage characterization.

To circumvent this limitation, many studies use approaches other than relying on the expression of single cytokines, such as the evaluation of polyfunctional cells (that is, cells expressing more than one cytokine at once). For example, the increased frequencies of IFNγ^+^IL-2^+^TNF^+^ CD4^+^ T cells were associated with improved anti-Mtb immunity^132^. Other studies have analyzed T cells after co-culturing them with other cell types, such as CD103^+^ DCs isolated from infected C57BL/6 or BALB/c mice, which resulted in higher frequencies of CD4^+^ T cells expressing IFN-γ or IL-17^133^. Others have limited the analysis of such cytokines to smaller subpopulations; this way, IL-12p40^+^ within CD103^+^ DCs^134^ and IL-2^+^ within KLRG1^−^ Mtb-specific CD4^+^ T cells^114^ were identified as infection and protection markers, respectively. Cytokines have also been useful for defining Mtb-specific cell populations where surface markers can be evaluated. The most notable example is the maturation marker CD27, which is expressed on the cell surface. In mice, CD27^low^ populations in IFN-γ^+^ CD4^+^ T cells were identified as a correlate of lung-homing properties and pulmonary TB activity^135^. These results have already been validated in human patients with TB in several independent studies^136,137^.

#### Novel mouse models

Because host genetic heterogeneity can greatly impact the outcome of Mtb infection, and given that common laboratory strains are inbred mice with identical genome, the TB field is currently focusing on two mouse models with more diverse genetic backgrounds: the Collaborative Cross (CC) and the Diversity Outbred (DO)^138–140^. The CC mice are a set of recombinant mice derived from five inbred laboratory strains (including C57BL/6) and three wild-derived lines. The founder strains are genetically distinct, and therefore the descendant mice retain a substantial portion of this genetic diversity as individual inbred lines. This model introduces genetic heterogeneity, while enabling reproducibility. Several studies have used CC mice to study how host–Mtb genetic interactions underlie susceptibility to TB^141,142^. A 2023 study identified several CC mouse strains that are naturally resistant or susceptible to Mtb infection when compared with the standard C57BL/6 mouse^143^. Interestingly, different CC mouse strains can also generate different responses to BCG vaccination, with some providing protection, while others do not^143^. Protection seems to be linked to CD4^+^ Th1/Th17 cells polyfunctionality, including elevated number of Forkhead Box P3 (Foxp3)- or RAR-Related Orphan Receptor Gamma T (RoRγT)-expressing or IL-17-producing CD4^+^ T cells, or decreased activated CD4^−^CD8^−^ T cells expressing T-box transcription factor (T-bet)^143^.

The DO mice take the CC diversity strategy a step further while using the same eight founder strains. They retain the same set of genetic variants as CC mice, but a rotational breeding maximizes heterozygosity within the population^144^. Upon BCG vaccination followed by Mtb infection, DO mice showed all possible phenotypes, with some mice exhibiting minor lung pathology accompanied by low bacterial burden, and others progressing quickly to uncontrolled disease^140^. The route of BCG vaccination (intravenous over intradermal) was also essential for enhanced protection^145^. DO mice were useful to study biomarkers associated with TB progression; initial studies showed a moderate accuracy for CXCL1, CXCL2 and CXCL5 expression in lung neutrophils^126^, of which CXCL1 (together with matrix metallopeptidase 8, MMP8) was further validated as a promising and translationally relevant biomarker candidate using machine learning approaches applied to multidimensional data^94^. The DO mouse model has also been used in blood transcriptomic analyses to show upregulation of *Parp9*, *Parp10* and *Parp14* (encoding ADP ribosyltransferases) during Mtb infection^146^ and in lung transcriptomic analyses to identify immune correlates of TB disease that are shared with macaques and already present in a 16-gene signature in humans^147^.

Overall, the CC and DO mouse models display substantial heterogeneity in their immune responses against BCG vaccination/Mtb infection, which opens the door to using these models to identify biomarkers, COP or susceptibility to Mtb infection and narrow down the mechanisms underlying such differences. These mouse models are designed to provide a more representative scenario of genetically diverse human populations that classical inbred lines are unable to capture.

### Small mammals: rats

Experiments using rats (*Rattus norvegicus*) share many of the advantages seen in experiments using mice, with the benefit that rats allow larger and more frequent blood collection. However, when it comes to TB studies, rats remain the least developed of the vertebrate models^148^. Previously thought to be resistant to Mtb infection^149^, it is now known that direct infection with the bacterium results in pathology in rats. Similar to the mouse model, infection outcomes vary widely depending on the inoculation method, rat strain and inoculation dose^150^. The TB model has been successfully applied with granuloma formation in the main rat breeds, such as Wistar, nude, American cotton and Lewis, among others^150^. However, human TB pathology is not fully recapitulated in most of them as they lack lesions that liquefy^151^. The past decades of TB research have not seen many reports of rat studies, and these focused on drug efficacy and safety rather than on biomarker discovery^152,153^. Notwithstanding, this model has provided valuable information on early phase biomarkers such as ED1 and OX62, and confirmed the involvement of inflammatory-associated molecules such as IFN-γ, IP-10 (IFN-γ-induced protein), TNF and iNOS (inducible nitric oxide synthase) with Mtb infection and/or TB pathology^154,155^.

### Small mammals: guinea pigs

The Hartley strain is the most used strain of guinea pig (*Cavia porcellus*) for modeling Mtb pulmonary infection. Guinea pigs are susceptible to low-dose infection (1–10 colony-forming units) and rapidly develop TB disease; therefore, they have provided valuable opportunities to investigate biomarkers during disease and after vaccination^156–158^. The disease observed in Mtb-infected guinea pig recapitulates acute infection in humans, showing many shared features^159,160^, including an immune response to infection characterized by the recruitment of innate and adaptive immune cells into the lungs and the development of granulomatous lesions^161^.

Past investigations of immunity include skin testing using purified protein derivate^162^. Subsequently, the identification and cloning of cytokines and chemokines provided the basis for future work to investigate immune responses to infection and vaccination^163–167^. Characterization of the immune response of guinea pigs to infection demonstrated T cell activity in the lung^168^ and production of Th1 cytokines by lung, spleen and peritoneal cells^162,169–173^ upon mycobacterial challenge. Due to the limited number of validated laboratory reagents, blood cytokine measurements to determine the immune status of the infected animals have relied on mRNA fold induction using reverse-transcription polymerase chain reaction after ex vivo stimulation of peripheral blood mononuclear cells (PBMCs). TB immunopathogenesis studies have relied predominantly on the analysis of infected organs after necropsy, before the development of validated flow cytometry reagents enabled cellular phenotyping during infection in guinea pigs. PBMCs have also been used in a mycobacterial growth inhibition assay for determining the capacity of immune cells from vaccinated guinea pigs to kill Mtb^174^. These studies have demonstrated that the mycobacterial growth inhibition assay could be used as a functional biomarker for determining vaccine immune responses. Analysis of blood metabolites after infection with and without prior BCG vaccination indicated that the liver enzymes alanine transaminase and alkaline phosphatase may provide reliable biochemical parameters for assessing vaccine efficacy^158^.

Overall, guinea pigs have provided a reliable model for assessing immunological markers that can be translated to humans. However, their immune toolkit is very limited compared with similar mammal models such as the mouse or rabbit. Increasing the number of laboratory reagents will help expand the use of this model in the field. Other limitations include the absence of available mutants for full or conditional knockout studies.

### Small mammals: rabbits

Rabbits (*Oryctolagus cuniculus*) are commonly used in TB research, representing a robust model to study TB in its different stages (latent, reactivation and active). A major advantage of this model is that it generates necrotic and/or liquefactive lesions resembling human granulomas upon infection with mycobacteria^175,176^. The course of Mtb infection in rabbits is well described. A report showed that, after aerosol infection with the Mtb strain CDC1551, the bacterial burden in the lungs of infected rabbits increased for the first 4 weeks before gradually decreasing thereafter. At 12 weeks post-infection, no bacteria were detected in the lung; however, subsequent immune suppression led to resumed bacterial growth, thus showing that latency rather than sterilization had been achieved^177^. This model is therefore useful to investigate the cellular and molecular networks operating in LTB. In addition, the same report showed that Mtb CDC1551-infected rabbits spontaneously cleared the bacteria after infection, which was associated with the downregulation of IL-1β, IL-8 and CCL2 and a decrease in lung immunopathology, associated with the downregulation of *MMP9*, *MMP12*, *MMP13* and *MMP14*^177^, providing molecular cues on immune alterations possibly associated with Mtb clearance. Subsequently, other studies explored the rabbit as a model to unveil host biomarkers of active TB. Mtb HN878-infected rabbits recapitulate the active disease phenotype, showing an increase of the lung bacterial burden in the first 4 weeks after infection, together with extensive necrosis in granulomas^178^. Furthermore, Mtb HN878-infected rabbits presented Rv20131c, Rv0934, Rv1860, Rv1886c and Rv2875 antigens, a profile that is notable given its similarity to that observed in humans with active disease^178^. A study comparing aerosol infections of rabbits with Mtb CDC1551 (LTB model) and HN878 (active disease model) revealed differential alterations in adipokine levels in adipose tissue and lungs in these models. Whereas LTB rabbits exhibited higher expression of adiponectin and PPARγ in the adipose tissue, rabbits with active disease had higher levels of adiponectin in the lungs^179^. The rabbit model of Mtb aerosol infection has proven to be a valuable and powerful model to assess drug penetration and/or distribution into different types of tubercular lesion^180–184^ and to test host-directed therapies^185,186^ as well as novel anti-TB drugs^187^.

As an alternative to aerosol infection, a skin-based infection model was also developed in rabbits. In this model, rabbits were injected intradermally with BCG, resulting in liquefied and ulcerative lesions on the skin, comparable to those found in the lungs^188^. This model has been used to evaluate the virulence of mycobacteria^189^ and the effects of immunomodulators on the lesions^188^. This model also serves as a platform to evaluate the protective efficacy of novel TB vaccines^190^. Following another strategy, rabbits have been sensitized with multiple subcutaneous injections of heat-killed *M. bovis*. This model was then used to record cavitary progressive disease by positron emission tomography/computed tomography (PET/CT) scans, finding that the uptake of [^18^F]-fluorodeoxyglucose correlated with inflammation and CFU burden, which makes this technique more sensitive for predicting cavitary disease than the CT scan technique^191^. In addition, the rabbit has also been extensively used in the research of extrapulmonary forms of TB such as spinal TB^192^. In this model, the decrease in inflammatory markers MCP-1 and NK-kB^193^ or TNF and CRP^194^ was associated with effective treatment with chemotherapy or medical devices. Collectively, these examples clearly show that rabbits are a great tool not only to deepen our knowledge of TB pathogenesis, but also to guide the development of new clinical tools for TB diagnosis and prevention. Some limitations of the model include its lower susceptibility to Mtb, as well as higher cost and greater space requirements compared with its smaller mammal counterparts.

### Large mammals: bovine and caprine models

Cattle and goats have natural susceptibility to *M. bovis* and *Mycobacterium caprae*, enabling the study of the same host–pathogen tandem that occurs in natural settings. The main features of pulmonary TB in experimentally challenged ruminants are similar to those found in human active TB. In the early stages, the animals show interstitial granulomas with abundant foamy macrophages and granulocytes that progressively accumulate more lymphocytes and multinucleated giant cells and develop necrosis, mineralization and fibrous capsule^195^. In advanced stages, these caseous-necrotizing granulomas may undergo liquefaction and form cavitary lesions, especially in goats^196^. However, the transition from infection to active disease and the occurrence of latent infections in these species are unclear^197^. Immune responses to infection rely primarily on antigen-specific CD4^+^ Th1 cells and the release of IFN-γ^196,198^, but CXCL10, CXCL9, IP-10 and IL-22, which are also specifically expressed in TB-infected animals, may be undetectable by IFN-γ release assay^199–201^. Immunomarkers such as the number of antigen-specific IFN-γ-producing PBMCs and increased expression of IL-17A by CD4^+^ and CD8^+^ cells have been associated with the severity of TB pulmonary lesions^202,203^. Conversely, antigen-specific IFN-γ-producing memory cell subsets, identified by flow cytometry or cultured enzyme-linked immunospot assays, are valuable predictors of vaccine efficacy, as they expand in vaccinated animals and inversely correlate with lung pathology following experimental challenge with the Mtb complex^204–207^. Vaccination of calves with BCG also induces trained innate immunity in circulating monocytes, enhancing their production of pro-inflammatory cytokines^208^. Although humoral immunity is usually detected at advanced stages of the infection, early antibody IgG levels to the MPB83 antigen (MPT83 in Mtb) were directly associated with burden^196,209^. Finally, plasma metabolic fingerprint using benchtop nuclear magnetic resonance spectroscopy has recently been shown to be an affordable new tool to differentiate TB-infected from uninfected animals^210^.

In summary, research on new vaccines and diagnostic tools can benefit from large animals, such as bovine and caprine models, because they share similarities with human TB immunopathology. However, experimentation on these and other larger mammals is complicated by ethical concerns and the requirement of expensive animal housing facilities and specialized personnel. In addition, these models have been developed using species different from Mtb for experimental challenge, making it harder to translate findings to humans compared with the following large animal model.

### Large mammals: NHPs

NHPs provide high-end TB models due to their phylogenetic, pathophysiological and immunological similarity to humans^211–214^. They have been instrumental in enhancing TB research and the development of new therapies, by accelerating the evaluation of clinical vaccines or drugs, designing improved candidates and optimizing formulation, dose and treatment regimen^215^.

NHP used in TB research include rhesus macaques (*Macaca mulatta*)^216,217^, cynomolgus macaques (*Macaca fascicularis*)^218,219^ and marmosets (*Callithrix jacchus*)^220,221^. The macaque models are valuable due to their similarity to human TB. One of the key features of the model is the formation of classical caseous tuberculous granulomas^211,212,222^; they also develop general human-like symptoms and mount a detectable peripheral adaptive immune response to Mtb infection at 3–5 weeks post infection, similar to humans^223–225^. Studies have reported that different macaque species and different genetic types or variations can naturally respond differently to disease, while environmental conditions in these outbred species may further contribute to diversity of TB disease manifestations upon experimental infection^226–231^. Varying the virulence of the Mtb challenge strain and/or dose also yields a range of phenotypes from acute progressive to disease-tolerant, latent-like infection, a state characterized by the absence of clinical symptoms, a positive TST result and/or Mtb-antigen specific T cell responses^230,232,233^. In 2020, intravenous injection of the standard BCG vaccine was provided to rhesus macaques for the prevention/limitation of Mtb infection phenotypes, yielding relevant contrast in the outcome of infectious challenges and allowing the identification of (vaccine-induced) correlates of protective TB immunity^216^. PET/CT in NHP TB studies is a valuable translational tool for longitudinal monitoring of TB disease, predicting reactivation of LTB and characterizing the organizational structure of granulomas^226,234,235^.

NHPs enable the investigation of not only peripheral but also local host responses against vaccination or infection under time-controlled conditions. IFN-γ^216,236^, TNF^237^, IL-17^224^, CD4^+^ T cells^238^, CD8^+^ T cells^239^ and NOS^240^ in macrophages have been shown to have a critical role in protection against Mtb infection in macaques. Furthermore, sterile granulomas have been shown to harbor higher frequencies of T cells producing Th1 (IFN-γ, IL-2 and TNF) and/or Th17 (IL-17) cytokines compared with nonsterile granulomas^236^. In addition, the characterization of granulomas in macaques provides an opportunity to understand COP in relation to the establishment of Mtb latency. In macaques, indoleamine 2,3-dioxygenase inhibits the access of lymphocytes to the central region of the granulomas, thereby making it a marker that can be targeted to induce protection^241,242^.

In the past decade, there has been growing interest in studying innate immune signatures, including trained innate immunity, in the context of vaccine-induced protection^243–245^. NHP studies have shown the induction of functional antigen-specific antibody subclasses locally, including IgA, in association with protective vaccination regimens. Interestingly, IgM titers have been correlated with reduced bacterial burden following intravenous BCG immunization^38,213,245,246^. In addition to antibodies detected by flow cytometry or immunohistochemistry techniques, single-cell RNA sequencing of lung tissue from rhesus macaques with latent versus active TB demonstrated that NK cells are a protective marker during Mtb latency^247^. The application of omics to NHP models to study TB and TB/HIV pathogenesis during the early infection phase can lead to the discovery of new biomarkers of protection in an unsupervised and unbiased manner^248–250^.

Furthermore, the macaque model can be used to study TB/HIV by co-infecting the animals with Mtb and simian immunodeficiency virus (SIV)^242,251,252^. SIV and Mtb co-infection results in massive depletion of CD4^+^ T cells in macaques, similar to TB/HIV co-infection in humans and in reactivation of LTB^232,233^. This model presents a unique opportunity to test novel therapeutic and vaccine approaches, which is not feasible in other animal models. In addition, work in the co-infection model has shown that treatment with antiretroviral therapy and/or anti-TB therapy may fail to reconstitute all Mtb-specific T cells in the lung compartment^226,232^. Hence, this model can be extended to elucidate the specific mechanisms leading to worsening of disease and mortality, including virus-driven immune suppression leading to LTB reactivation and to model immune reconstitution inflammatory syndrome. As with the bovine and caprine models, investigations with NHPs are limited to selected studies owing to the enormous cost of their use, as well as space and installations requirements.

### Animal models for the study of NTM

Some of the abovementioned models have also been used to study NTM (species other than Mtb complex and *Mycobacterium leprae*). This is of substantial importance because these mycobacteria cause long-lasting, difficult-to-treat pulmonary and disseminated pathologies whose incidence has already surpassed that of Mtb in developed countries^253^. In this context, the identification of specific biomarkers for NTMs in animal models is essential to understand immunopathology; however, it is still challenging due to the difficulty of establishing the infection in vivo. Anemia and alterations in host iron metabolism, which are frequent complications of chronic infections involving mycobacteria, have been studied using in vivo models including mice intravenously infected with *M. avium;* in vivo studies have also investigated how the host metabolism modulates NTM infections^254–256^. Regarding *M. abscessus*, it has been challenging to find a suitable animal model to establish the infection. However, a mouse model of infection described in 2020 showed that daily administration of corticoids before and after intranasal infection (which allowed higher levels of bacterial loads in the lungs) made the animals more prone to pathology^257^. In addition, a 2022 study demonstrated the applicability of the adult zebrafish as a model of persistent *M. abscessus* infection, revealing differences in the immunopathogenesis induced by rough and smooth variants during granulomatous infection^258^. Zebrafish was also a suitable model for *M. fortuitum*^259^. The lack of animal models has been reported for *Mycobacterium kansasii*, which causes severe pulmonary disease that is indistinguishable from TB in human adults. However, a 2021 study established a model of *M. kansasii*-induced pulmonary pathology by intratracheal infection of C57BL/6 mice with highly virulent strains from clinical isolates^260^. Moreover, as detailed in other sections of this review, the study of host–pathogen interactions in different animal models infected with NTM species has contributed to our understanding of TB disease. This is the case for *M. marinum*,which has been used to study autophagosome formation during infection with Mtb or NTM in *D. melanogaster* (reviewed elsewhere^57^), as well as for *M. bovis* and *M. caprae* in cattle models. Despite their drawbacks and practical limitations, different animal models of NTM infection have made a strong contribution to the field by improving our understanding of pathogenesis and guiding drug and vaccine development, not only for NTM infection but also for Mtb infection through mimicking studies^261^.

## Concluding remarks

TB still poses a major challenge for the scientific community. The complex pathogenesis and progression of TB seen in humans is differentially recapitulated in experimental in vivo models, where infection route, lesion type, organ manifestations and disease form and severity, among others, differ between models. As described in this Review, none of the currently available animal models recapitulates all aspects of the human disease; instead, they provide pieces of information that need to be assembled into an overall picture. Fortunately, the long list of well-defined animal models and vast number of available resources outweighs some of the limitations associated with each model. In this Review, we have described the advantages of each animal model, explaining how they have contributed to advancing the field of TB biomarkers (summarized in Table 1). Altogether, the most promising biomarker might be in fact a combination of molecules, preferably those involved in inflammation (cytokines) and homing (chemokines). However, many challenges still need to be tackled. While detecting biomarkers of TB progression in latently infected patients is a priority, most animal models of TB latency exhibit poor reproducibility, and the mechanisms driving the transition to active disease remain largely unclear. There is also limited literature on biomarkers that predict long-term recurrence and/or relapse of TB, probably due to the long time required by the models to achieve similar scenarios. These limitations hinder vaccine and drug evaluation studies and should therefore be a research priority in the field.

Investigation on immune biomarkers will remain a priority in the TB field for the foreseeable future, as robust biomarkers of immune protection are key for vaccine development, while those associated with treatment response could help improve antibiotic regimens. The challenges inherent to human studies are unlikely to disappear. Despite major efforts and advances to replace animal models with human-based two- and three-dimensional organoid models, the lack of full organ structure and physiological microenvironment limits the ability to rely solely on in vitro systems to advance our understanding of disease pathogenesis. Mtb remains a paradigm of complexity among human lung pathogens; therefore, complementing in vitro studies with animal models is essential for assessing therapies and vaccines, as well as for discovering biomarkers. As science advances, animal models of TB will undoubtedly become more refined, generating data that are not only more accurate and reproducible but also produced more quickly and safely.
